# Cilengitide-Induced Temporal Variations in Transvascular Transfer Parameters of Tumor Vasculature in a Rat Glioma Model: Identifying Potential MRI Biomarkers of Acute Effects

**DOI:** 10.1371/journal.pone.0084493

**Published:** 2013-12-23

**Authors:** Tavarekere N. Nagaraja, Madhava P. Aryal, Stephen L. Brown, Hassan Bagher-Ebadian, Tom Mikkelsen, James J. Yang, Swayamprava Panda, Kelly A. Keenan, Glauber Cabral, James R. Ewing

**Affiliations:** 1 Department of Anesthesiology, Henry Ford Hospital, Detroit, Michigan, United States of America; 2 Department of Neurology, Henry Ford Hospital, Detroit, Michigan, United States of America; 3 Department of Radiation Oncology, Henry Ford Hospital, Detroit, Michigan, United States of America; 4 Department of Diagnostic Radiology, Henry Ford Hospital, Detroit, Michigan, United States of America; 5 Department of Neurosurgery, Henry Ford Hospital, Detroit, Michigan, United States of America; 6 Public Health Sciences, Henry Ford Hospital, Detroit, Michigan, United States of America; 7 Department of Physics, Oakland University, Rochester, Michigan, United States of America; 8 Department of Neurology, Wayne State University, Detroit, Michigan, United States of America; University of Regensburg, Germany

## Abstract

Increased efficacy of radiotherapy (RT) 4-8 h after Cilengitide treatment has been reported. We hypothesized that the effects of Cilengitide on tumor transvascular transfer parameters might underlie, and thus predict, this potentiation. Athymic rats with orthotopic U251 glioma were studied at ~21 days after implantation using dynamic contrast-enhanced (DCE)-MRI. Vascular parameters, *viz*: plasma volume fraction (*v*_*p*_), forward volume transfer constant (*K* ^*trans*^) and interstitial volume fraction (*v*_*e*_) of a contrast agent, were determined in tumor vasculature once before, and again in cohorts 2, 4, 8, 12 and 24 h after Cilengitide administration (4 mg/kg; N = 31; 6-7 per cohort). Perfusion-fixed brain sections were stained for von Willebrand factor to visualize vascular segments. A comparison of pre- and post-treatment parameters showed that the differences between MR indices before and after Cilengitide treatment pivoted around the 8 h time point, with 2 and 4 h groups showing increases, 12 and 24 h groups showing decreases, and values at the 8 h time point close to the baseline. The vascular parameter differences between group of 2 and 4 h and group of 12 and 24 h were significant for *K* ^*trans*^ (p = 0.0001 and *v*_*e*_ (p = 0,0271). Vascular staining showed little variation with time after Cilengitide. The vascular normalization occurring 8 h after Cilengitide treatment coincided with similar previous reports of increased treatment efficacy when RT followed Cilengitide by 8 h. Pharmacological normalization of vasculature has the potential to increase sensitivity to RT. Evaluating acute temporal responses of tumor vasculature to putative anti-angiogenic drugs may help in optimizing their combination with other treatment modalities.

## Introduction

In treating brain tumors, the importance of optimizing the timing and sequence of standard therapies such as radiotherapy (RT) with newer anti-angiogenic drugs is being increasingly recognized [1,2]. These insights are supported by data showing that several experimental anti-angiogenics are effective in combination with RT in a specific temporal sequence [2–5]. 

Angiogenesis has been long identified as a potential target to reduce the growth of solid tumors [6,7]. Integrins, a class of transmembrane receptors that facilitate cell-cell and cell-extracellular matrix interactions present a novel target in the efforts to inhibit the growth of tumor vasculature [8]. Specifically, the α_v_β_3_ and α_v_β_5_ integrins recognize the arginine-glycine-aspartate peptide domain of extracellular ligands that regulate tumor migration and angiogenesis via formation of membrane focal adhesions [9,10]. Attempts are being made to inhibit integrin binding and angiogenesis via blockage of this peptide domain by either primary antibodies or linear peptides [11]. Of these, Cilengitide, a cyclic Arg-Gly-Asp-DPhe-NMe-Val (RGD) peptide, showed promise in inhibiting integrin-mediated angiogenesis in solid brain tumors such as glioblastoma [12,13]. MacDonald et al. [13] observed site-specific effects of Cilengitide when it was tested in orthotopic (brain) and heterotopic (subcutaneous) implantations of U87 glioma in nude mice. Daily systemic treatment with Cilengitide reduced the size of brain tumors and increased survival without affecting the subcutaneous tumor in the same animals. Similar effects were observed by Yamada et al. [14] who reported U87 glioma tumor size reduction and decreases in tumor cell proliferation and blood vessel growth after Cilengitide treatment. Further confirming its utility in treating brain tumors, Cilengitide was also shown to be an efficient adjuvant to RT in multimodal cancer treatments [1,5]. As a result, Cilengitide entered Phase III clinical trials for the treatment of gliomas [11,15]. The results of this trial, the CENTRIC study, were recently reported. Despite a good safety profile and a slight, but not statistically significant, increase in progression-free survival (13.5 months with Cilengitide vs. 10.7 months without), Cilengitide treatment in addition to standard care with RT and temozolomide was not found to extend life expectancy in newly diagnosed glioblastoma [16].

Nonetheless, integrin inhibitors, including Cilengitide, have exhibited some unique temporal effects in combination with RT. An integrin inhibitor, S247, another RGD peptide antagonist of α_v_β_3_ integrin, showed excellent synergy with external beam radiotherapy [3]. A similar finding was reported in that a single injection of Cilengitide given between 4-12 hours before RT had a synergy with RT in a U251 glioma model *in vivo*. In this latter study, when tested using *in vitro* preparations, Cilengitide amplified the cytotoxic effects of radiation on endothelial cells, but not on U251 cells [5]. Such effects could not be explained solely by its integrin blocking and anti-angiogenic actions, suggesting that Cilengitide and similar drugs may have some specific short-term effects on the tumor vasculature that magnify RT efficacy in a time-sensitive manner. 

It has been suggested that vascular normalization, including lowered permeability, is one of the factors determining RT efficacy [17]. Since, in the brain, tumor blood vessels are often leaky in a milieu that is otherwise characterized by tight junctions, we hypothesized that short term effects of Cilengitide included altered transvascular transfer parameters of the tumor vasculature. The model employed was that of a U251 cerebral glioma in the athymic rat; the technique used to evaluate vascular parameters was dynamic contrast-enhanced magnetic resonance imaging (DCE-MRI) with a model selection based quantitative pharmacokinetic analysis [18–21].

## Materials and Methods

### Ethics statement

This study was carried out in strict accordance with the recommendations in the Guide for the Care and Use of Laboratory Animals of the National Institutes of Health. The experimental protocol was approved by the Institutional Animal Care and Use Committee (IACUC) of the Henry Ford Hospital (IACUC #1150). Tumor implantations were performed under ketamine and xylazine anesthesia and MRI under isoflurane anesthesia; all efforts were made to minimize animal discomfort and suffering.

### The orthotopic U251 tumor model

Athymic, nude rats (~8 weeks old; Charles River, Wilmington, MA) were implanted intracerebrally as follows: animals were anesthetized with intramuscular 80 mg/kg ketamine and 15 mg/kg xylazine. The surgical zone was swabbed with Betadine solution, the eyes coated with Lacri-lube and the head immobilized in a small animal stereotactic device (Kopf, Cayunga, CA). A burr hole 2.5 mm lateral to the right of Bregma was made in the skull. A 10 μl capacity Hamilton syringe with a 26 gauge needle containing U251MG tumor cells (5x10^5^ in 10 μl of phosphate buffered saline) freshly harvested from log phase growth was lowered to a depth of 3 mm, raised to back to 2.5 mm to create a tissue pocket. Tumor cells were then deposited into this pocket at a rate of 0.5 μl/10 s until the entire volume was inoculated. The syringe was then slowly withdrawn, the burr hole sealed with sterile bone wax and the skin sutured. Tumors in animals implanted following this technique grew to 3 to 4 mm diameter by about 3 weeks post-implantation.

On day 21, each animal was anesthetized (isofluorane 4% for induction, 0.75 to 1.5% for maintenance, balance N_2_O:O_2_=2:1) and allowed to spontaneously respire. A tail vein was cannulated for the administration of MR contrast agent (MRCA). Body temperature was maintained constant (37 °C) with a warm air supply monitored via an intrarectal type-T thermocouple. 

### MRI studies

All studies were performed in a Varian (now Agilent Technologies, Santa Clara, CA) 7 Tesla, 21 cm bore system with a Direct Drive spectrometer and console. Gradient maximum strengths and rise times were 250 mT/m and 120 ms. All MRI image sets were acquired with a 32x32 mm^2^ FOV. Three weeks after implantation with U251 cerebral glioma cells two DCE-MRI studies were performed 24 hours apart. Cilengitide (Merck; 4 mg/kg; intraperitoneal; N = 6-7 per cohort) was administered before the second study at the following times: 2, 4, 8, 12, and 24 hours. An image set consisting of T_1_, cerebral blood flow (CBF) and diffusion weighted imaging (DWI) were part of the protocol to differentiate and localize the tumor from the surrounding normal brain tissue [22]. 

The image sets were acquired by means of a dual-echo gradient echo (2GE) sequence, the “mgems” sequence in the Agilent VNMRj library. The 2GE sequence acquired 150 image sets at 4.0 sec intervals: (matrix = 128x64, three 2.0 mm slices, NE= 2, NA=1, TE1/TE2/TR = 2.0/4.0/60 ms). Bolus injection of a magnetic resonance contrast agent (MRCA) (Magnevist; Bayer, Wayne, NJ; 0.25 mmol/kg) was performed by hand push at the 15th image. Prior to the 2GE sequence, and immediately after, two Look-Locker (LL) sequences (matrix 128x64, five 2.0 mm slices, NE=24 inversion-recovery echoes, TR=2000 ms) were run so that a voxel-by-voxel estimate of longitudinal relaxation time (T_1_) in the tissue could be made for pre- and post-CA administration. Prior to the first LL sequence, a fast spin-echo arterial spin-labeled pulse sequence (matrix 128x64, TR=1500ms, TE=24.04ms, single 1.0 mm slice) was run to generate the CBF maps. Following the post-contrast LL sequence, a Pulsed Gradient Spin-Echo DWI sequence (matrix 128x64, thirteen 1mm slices, TR=1500ms, TE=40 ms, NE=1, b-values = 0, 1217 s/mm^2^, gradient amplitude=107 mT/m, gradient duration = 10 ms) was run to generate a parametric map of apparent diffusion coefficient (ADC). Similarly, high-resolution T_1_-weighted images were acquired pre- and post-CA with the following parameters: matrix size 256x192, 27 slices, 0.5 mm thickness, no gap, NE = 1, NA = 4, TE/TR = 16/800 ms.

Image analysis was performed using a previously developed model selection paradigm [18,20,23] that generates maps of brain regions with: i) only plasma distribution volume, *v*_*p*_ (essentially normal vasculature with no leakage; Model 1); ii) *v*_*p*_ and forward volume transfer constant, *K* ^*trans*^ (some parts of tumor with leakage, but with only blood-to-brain influx; Model 2); or iii) *v*_*p*_, *K* ^*trans*^ and extravascular extracellular distribution volume, *v*_*e*_ (highly leaky vessels with measurable backflux; Model 3) [18,19]. Quantitative maps of two separate sets of these parameters for the MRCA from Model 3 regions were made for the ‘Test’ and ‘Retest’ imaging sessions.

### Histopathology

After the second MRI study, the animals were deeply anesthetized with ketamine-xylazine cocktail and transcardially perfused with normal saline followed by 4% paraformaldehyde. After the brains were carefully removed from the skull, they were post-fixed in same fixative overnight. Coronal sections through the tumor were obtained using a rat-brain matrix (Activational Systems, Inc., Warren, MI). The brain tissue was processed using a VIP Tissue Tek Processing center, prior to embedding. Seven-micrometer-thick sections were cut from the block representing the MR imaging slice and placed on Superfrost Plus (Fisher Scientific) slides. It contained the largest tumor area and sections five through seven were stained with hematoxylin and eosin (H&E). Sections one through four in the block were stained for vWF as described below. 

### Von Willebrand factor immunohistochemistry

Slides were dried at 37° C for 2 hours, cleared in xylene and rehydrated in an ethanol series. Antigen retrieval was performed using proteinase K prior to endogenous peroxidase and normal serum blocking. Sections were incubated overnight in primary antibody vWF (1:500, Millipore USA, Billerica, MA), and followed by rabbit IgG (Vector, Burlingame, CA) and avidin-biotinylated horseradish peroxidase complex (ABC, Vector, Burlingame, CA). Chromogen reactions were developed on sections with diaminobenzidine (DAB; Vector Laboratories, Burlingame, CA) and then counterstained with hematoxylin.  A Nikon Eclipse E800 Microscope equipped with ACT1C software was utilized for image collection. The number of vascular segments staining positive for vWF per field were counted at 10X magnification in selected regions of interest (ROI) spanning the core, body and periphery of the tumor and in the contralateral caudate-putamen.

### Data analysis

All data are reported as mean ± standard deviation. For the MR parameters, only those regions satisfying the conditions for Model 3, i.e. in which all the 3 parameters of the standard model, *viz*., *v*_*p*_, *K* ^*trans*^ and *v*_*e*_, could be analyzed, were included in the comparisons. Following convention, the initial MR data (prior to Cilengitide treatment) were defined as ‘test’ and the 2^nd^ set as ‘retest’ (post-treatment). The definition of a parameter difference between test and retest is the percentage difference between ‘value test’ and ‘value retest’. Since Cilengitide was administered before the second study, we evaluated the effect of Cilengitide using the paired t-test at each time point. In addition, boxplots were used to illustrate the distribution of test-retest differences at each time point. To compare the time effect of administering Cilengitide, one-way analysis of variance (ANOVA) was used for each parameter. Based on visual examination of the boxplot and the F-test from one-way ANOVA, the contrast comparing 2 to 4 h treatment group and 12 to 24 h group was identified and tested. The vascular segments staining for vWF were counted in tumor periphery, body and core using sections at 10X magnification in 5 fields. The number of segments from the 5 treatment groups was compared to that from an untreated group for noting any differences.

## Results

**Figure 1:**
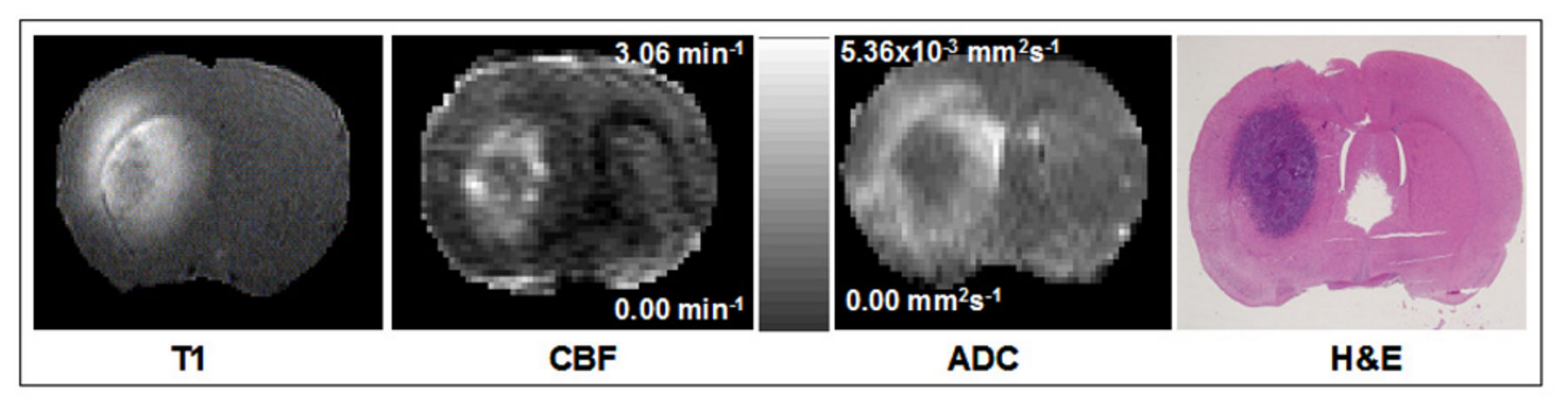
An example from one experiment to illustrate the experimental localization of tumor. A series of high resolution T_1_, cerebral blood flow (CBF) and apparent diffusion coefficient of water (ADC) maps and the corresponding H&E stained brain section are shown. Note the anatomical agreement in the position and distribution of the tumor mass between MRI and histopathology. The scale bar between CBF and ADC maps is common to them both, but has different scaling as indicated in the range for each.

Signs of a space-occupying tumor such as distress were just being evident in the animals by 3 weeks post-implantation. The MR images usually showed a generally oval tumor of about 3-4 mm diameter with a well delineated perimeter within the caudate-putamen. An example is shown in Figure 1 illustrating the experimental localization of tumor. High resolution T_1_-weighted images showed a ring of hyperintensity around the tumor that corresponded with the well perfused tumor periphery seen on CBF maps. The ADC map showed hyperintensity beyond the periphery suggesting increased water content and edema. In addition, the H&E stained sections (Figure 1) confirmed the MRI observations on the location and extent of the tumor mass in all rats. A few rats also had tumor cells migrating along the needle track and forming a smaller mass toward the surface. A midline shift and slightly compressed lateral ventricles were observed in rats with larger tumors. 

**Figure 2:**
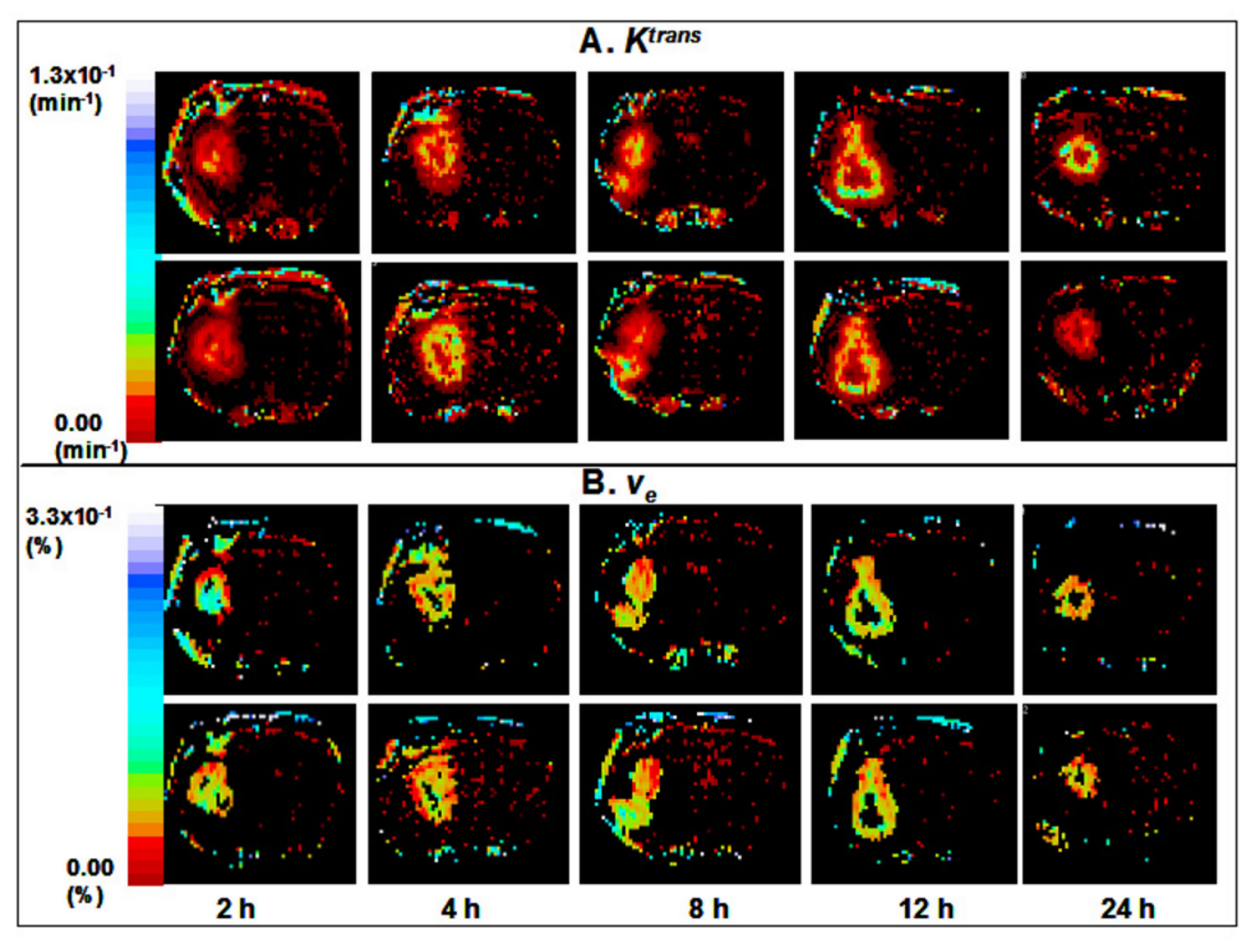
Representative sets of *K* ^*trans*^ maps (A) from five different experiments before and after Cilengitide treatment in each set. The times at the bottom refer to the time at which the drug was administered before the second MRI at 24 h and apply to both 2A and 2B. The top tier in each parameter is ‘Test’ and the bottom tier, ‘Retest’. A color scale bar is given in common to all maps for a given parameter. Note the apparent increases (increase in yellow and blue hues) at 2 (N = 7), 4 h (N = 6) after treatment and the decreases (decreases in yellow and blue hues) in *K* ^*trans*^ values at 12 (N = 6), 24 h (N = 6) after treatment. Representative sets of *v*_*e*_ maps from the same five experiments before and after Cilengitide treatment in each set are shown in B. The changes, however, are not that obvious as in Figure 2A.

**Figure 3:**
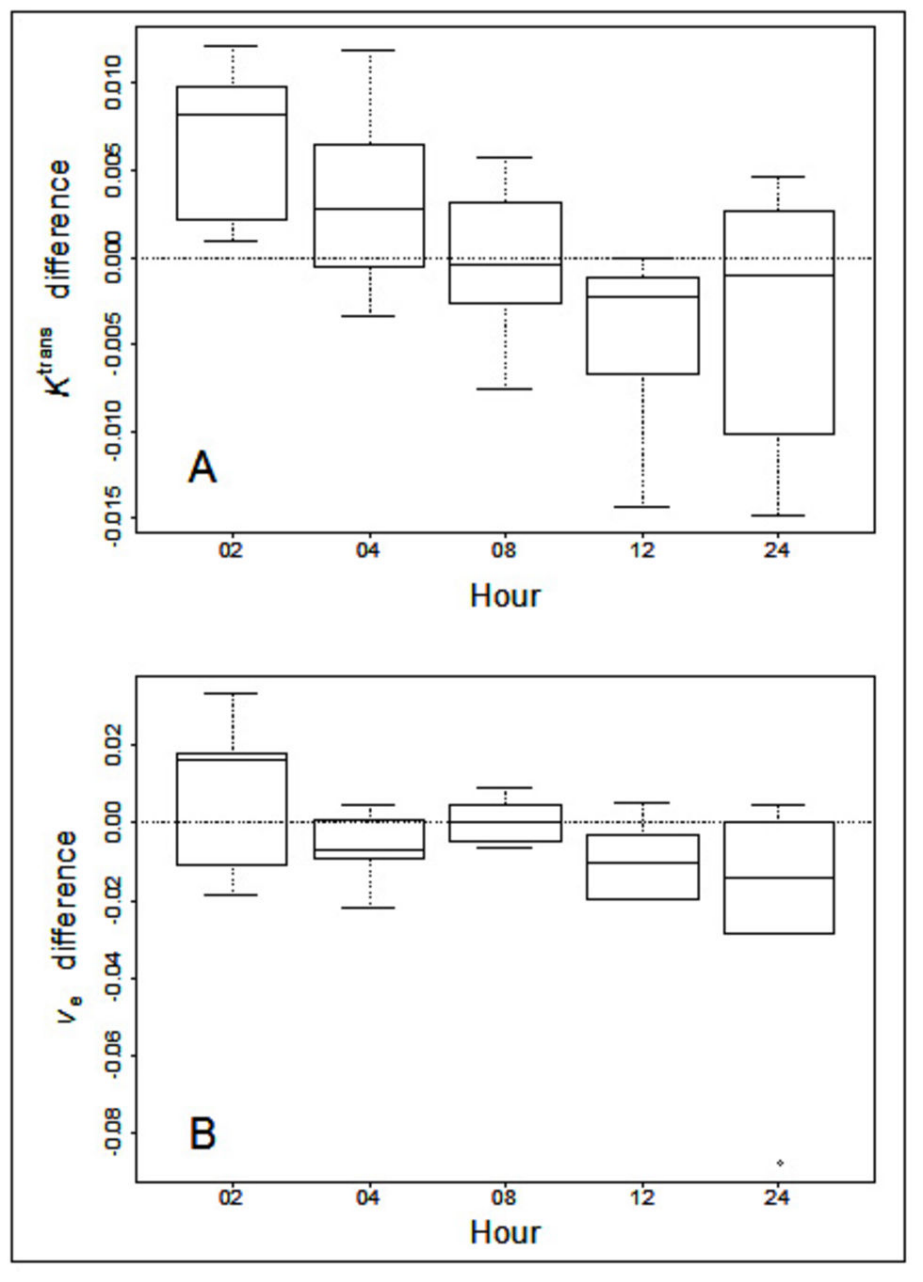
Boxplots of the data from Figure 2 for *K* ^*trans*^ (A) and *v*_*e*_ (B). Note the overall decreasing trend from 2 to 24 h after Cilengitide treatment with the 8 h (N = 6) points being close to 0% change from the pre-treatment values or baseline in A.

Maps of CBF revealed no significant changes between the different treatment groups (data not shown). Examples of *K* ^*trans*^ and *v*_*e*_ parameter maps from rats treated with Cilengitide at different times with respect to the 2^nd^ imaging session are shown in Figure 2A and 2B. The distribution of the *K* ^*trans*^ difference and *v*_*e*_ difference at each time point are presented in Figure 3. The upper panel shows that the *K* ^*trans*^ difference decreased as the hour increased. At hour 8, the *K* ^*trans*^ difference is distributed around 0% change. The F-test comparing before 8-h and after 8-h showed that the *K* ^*trans*^ differences were distributed differently before and after 8-h (p value = 0.0001). Therefore, in this study we showed that *K* ^*trans*^ differences were positive in the early hours and reached 0% around 8-h and decreased after that. Using the same analysis approach for the lower panel of Figure 3, we show that the *v*_*e*_ difference reached 0% change at 8-h and that *v*_*e*_ differences were significantly different before and after 8-h (p value = 0.0271). The summary statistics and p-values for the inter-group comparisons of MR parameters are given in Table 1.

**Table 1:** Comparisons between the group of 2 and 4 hour and the group of 12 and 24 hour for the difference between test and retest on variables of *K* ^trans^, *v*_*e*_ and *v*_*p*_.

*Variable*	*GROUP*	*N*	*Mean*	*Std Dev*	*p value*
*K^trans^ dif*	*2 and 4 hour*	*13*	*0.0050*	*0.0050*	*0.0001*
	*12 and 24 hour*	*12*	*-0.0039*	*0.0064*	
*v_e_ dif*	*2 and 4 hour*	*13*	*0.0005*	*0.0166*	*0.0271*
	*12 and 24 hour*	*12*	*-0.0164*	*0.0251*	
*v_p_ dif*	*2 and 4 hour*	*13*	*0.0016*	*0.0052*	*0.1916*
	*12 and 24 hour*	*12*	*-0.0006*	*0.0030*	

The means and standard deviations were calculated within each group. However, the p-value was derived using contrast of one-way ANOVA.

The temporal changes in *K* ^*trans*^ and *v*_*e*_ are observed in the boxplots shown in Figure 3. From the Figure 3, we observed that the time in which *K* ^*trans*^ and *v*_*e*_ reached 0% change occurred at 8-h. The inter-quartile intervals covered 0% change indicating there were no significant differences between test and re-test for *K* ^*trans*^ and *v*_*e*_. at 8 hours.

**Figure 4:**
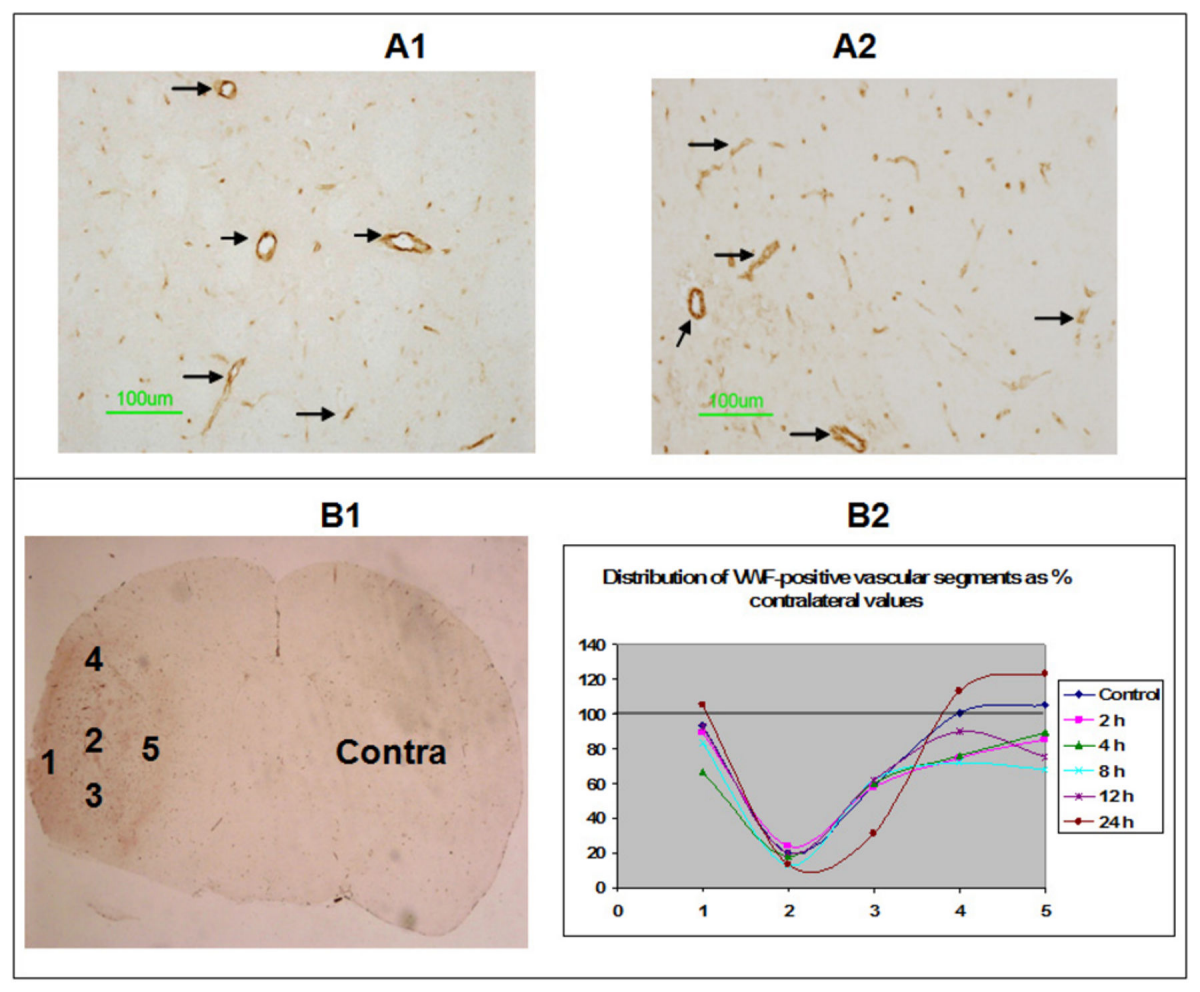
vWF-positive vascular segments, their visualization and quantification. Images from the contralateral hemisphere (A1) and a tumor periphery (A2) are shown at 20X magnification to exemplify the staining. The stained vasculature is indicated by arrows. Scale bar = 100 μm. Some examples of ROIs of interest 1-5 spanning the tumor along with the contralateral ROI (Contra) from a low magnification vWF stained coronal section are shown in B1. Distribution of the mean number of stained segments from each ROI expressed as % of the contralateral value is shown 4B. The numbers on abscissa indicate the ROI (shown in 4B) from which the measurements were made. The ordinate is the % contralateral value. Treatments regimens are color-coded in the graph. The values from the various treatments were not significantly different from untreated controls (Blue). Black solid line represents the 100% value from contralateral hemisphere.

Some examples of vWF-positive vascular segments are shown in Figure 4A1 and 4A2. These are at 20X magnification. The vascular segments are visible mostly as vessel cross sections stained dark brown by the DAB reaction over the lighter hematoxylin-stained background. The low magnification image of the coronal brain section shown in 4B1 indicates the various ROIs from which the segments were counted for quantification. They span the tumor core, body and periphery. One ROI from the corresponding contralateral hemisphere was also included in the counting. The distribution patterns of vWF-positive vascular segments along with their approximate positions on the coronal section for the respective ROIs are shown in 4B2. It can be seen that the number of vascular segments are always the lowest in the core than in other tumor regions or contralateral side. This was consistent keeping with previous reports of a necrotic center in the U251 glioma models. However, the number of vascular segments between the treatment groups was not significantly different for any ROI. 

## Discussion

The U251 orthotopic glioblastoma tumor model constitutively expresses the αvβ3 and αvβ5 integrins and can be employed to study the acute and chronic effects of agents that interfere with integrin actions [5,12,24]. The noninvasive measures of vascular parameters presented in this paper demonstrate that Cilengitide exerted specific short-term effects on blood-to-brain transfer parameters of *K* ^*trans*^, and on *v*_*e*_. Such effects were biphasic with a transient normalization point at post-treatment 8 h. The trend toward functional vascular normalization between 4 and 12 h post-treatment is significant since it coincided with the time of maximum therapeutic benefit when Cilengitide was combined with RT in the same tumor model [11]. 

Despite the presence of αvβ3 and αvβ5 integrins in both tumor- and endothelial-cells, preferential actions of Cilengitide on endothelial cells *in vitro* have been demonstrated. While it potentiated RT effects in endothelial cells, such effects were not observed in tumor cells [5]. These data imply that Cilengitide may exert some unknown actions on endothelial cells apart from its known effects on integrins. It is plausible that such actions may also be region-specific since MacDonald showed that Cilengitide treatment stunted the growth of orthotopic, but not heterotopic, U87 tumors [13]. Such actions may be a shared property of anti-angiogenic drugs since acute cerebrovascular perfusion alteration as a mechanism of action has been suggested for other anti-angiogenic agents such as an anti-VEGF molecule, SU11657 [25]. These authors investigated single, bi- and tri-modal therapy with A431 human tumor xenografts *in vitro* and *in vivo* and observed that tri-modal approach was superior to single and any combination of bi-modal therapy. A crucial aspect, however, was that in bi-modal therapy with SU11657, RT administered before SU11657 was less effective than after. The tumors treated with SU11657 also had less edema and lower interstitial fluid pressure after 10 days of treatment than untreated ones. The authors attributed these effects to the presumed normalizing of tumor vasculature by SU11657 which can reduce vascular leakage, a source of edema. 

Ceramide-mediated endothelial apoptosis by VEGF receptor-2 antagonist as a mechanism of increased radiosensitivity has also been shown [2], although the importance of this effect in RT effectiveness is the subject of current debate [26]. Observations by Maurer and co-workers also support the assumption of vasculature-specific actions of anti-angiogenics that act in concert with RT [27]. They reported that in clinically relevant concentrations, Cilengitide did not modulate sensitivity to either radiation or temozolomide treatment of human glioma cells *in vitro* and concluded that beneficial effects of Cilengitide *in vivo* may arise from some, yet unknown, changes in perfusion that served to promote drug delivery [27]. In the present study, direct *in vivo* evidence is provided for Cilengitide-induced tumor vasculature functional normalization, albeit transiently, in terms of blood-to-brain transfer indices at 8 h after administration. Given the plasma half life of about 20 minutes for Cilengitide, the mechanisms of this normalization are not clear. One possible mechanism of increasing the damage caused by RT may be the inhibition of radiation-induced Akt phosphorylation by integrin-blockers such as Cilengitide. However, its effects on tumor vascular permeability cannot be explained by this mechanism. Acute reductions in the number of vascular segments were not present at these times, but may become evident with longer treatment durations as seen with other drugs [25]. 

There is a need still to understand the region-specific effects of Cilengitide and other such drugs. Inhomogeneity of vascular permeability in tumors is known. Especially large tumors can have loci within them with small amounts of leakage with little backflux, and loci with significant leakage with measureable backflux. It should be noted that the ability to measure *v*_*e*_ in the present case also implies significant amounts of backflux, *k*_*ep*_, since *v*_*e*_ = *K* ^trans^/*k*_*ep*_. It is not clear yet whether Cilengitide preferentially acts upon these highly permeable microvessels [28,29]. Moreover, *K* ^*trans*^ is dependent on both CBF and vascular permeability. Since CBF changes were not observed, factors that affect permeability such as tight junction proteins may need to be studied. There is some evidence that leakage of blood-borne materials of up to 800 Da (Magnevist = 938 Da) is facilitated by alterations in the membrane protein claudin-5 [30], a component of tight junction strands; extravasation of larger moieties needs additional changes in other tight junction proteins such as occludin. Further studies are needed to determine whether these tight junction proteins are also the targets for Cilengitide-mediated acute functional vascular normalization. 

For exerting their anti-angiogenic effects, Cilengitide and other such drugs need to be administered chronically [31,32]. However, their acute actions and short-term effects on vasculature seem equally significant in planning a treatment regimen. In agreement with previous recommendations [2], evaluating the effects on vascular perfusion status at regular intervals during treatment may help determine optimal combination times with fractionated RT and chemotherapy, It is noteworthy in this context that tumor vascular normalization can be achieved by several different treatments and has varying implications depending on the treatment mode [17]. It can be transient as is the case with anti-angiogenics [33], but longer lasting when achieved by suppression of tumor cell oncogenes [34]. It may be due to drug-induced balance between pro- and anti-angiogenic factors [35] or ablation of leaky neovasculature [36]. Irrespective of the means, vascular normalization is believed to enhance RT effects and/or chemotherapy delivery via decreased hyper-permeability (lower *K* ^*trans*^), consequent attenuated tumor interstitial fluid pressure (TIFP) and increased tumor oxygenation [34]. Of the MR parameters assessed in the present study, *K* ^*trans*^ proved to be the most sensitive to treatment effects, an observation also made in several previous reports discussed below. Drugs that reduce brain edema with a single dose such as dexamethasone (8 mg/kg) have been shown to lead to a global reduction, a 40% reduction in *K* ^*trans*^ for Gadomer (a large experimental CA of 18 kDa; Schering, Germany) in vascular transfer parameters in a 9L glioma model [19]. An identical parameter for radiolabeled serum albumin measured by quantitative autoradiography also declined after dexamethasone treatment (8 mg/kg, given over 48 h) in a rat model of RG-2 glioma [37]. In rat P22 flank tumors, *K* ^*trans*^ was a reliable indicator of vascular status after treatment with Combretastatin, an anti-vascular agent [38]. Alterations in permeability parameters to small gadolinium analogs have also been shown to reflect treatment effects in human brain tumors [39]. Therefore, changes in *K* ^*trans*^ measured by DCE-MRI may be a powerful tool to evaluate vascular normalization by other putative anti-angiogenic drugs. Employing a small MRCA already in clinical use such as Magnevist, along with proper model selection criteria [20,23] can be of help in such evaluations. 

Our study, however, has some limitations that may restrict broader generalizations of the observations. A main one is the lack of demonstration of normalization of tumor milieu using other indicators such as reduction in tumor hypoxia. It is known that hypoxia renders the tumor tissue resistant to RT. Along with vascular permeability, whether reduction in hypoxia was also part of the mechanism of Cilengitide’s acute actions is not clear from the data and needs further investigations to clarify that aspect. Another shortcoming is the absence of demonstration of tumor vascular localization of α_v_β_3_ and α_v_β_5_ integrins in the model we have employed. There are evidences from published literature, however, that the U251 glioma model does express these integrins [5,12], which lend support to our interpretations. 

## Conclusions

In Phase III clinical trials, Cilengitide did not increase overall survival and progression-free survival in combination with standard therapy. However, its safety profile in combination with standard therapy was once again confirmed [16]. It is noteworthy that in other brain cancer models such as for meningioma, Cilengitide was effective in combination with RT in decreasing tumor volume [40]. Cilengitide is also reported to be brain-specific in its actions[13,40]. The present results suggest that apart from its known anti-angiogenic properties, Cilengitide may have other short-term and temporally dependent protective effects on tumor vasculature. It is known that inhibition of integrins leads to decreased interstitial pressure [8,41–43]. Thus, it may be necessary to further evaluate Cilengitde and other such integrin inhibitors for their effects on tumor hypoxia and tumor interstitial pressure to understand their mechanisms better. , 
